# Longitudinal changes in DTI parameters of specific spinal white matter tracts correlate with behavior following spinal cord injury in monkeys

**DOI:** 10.1038/s41598-020-74234-2

**Published:** 2020-10-14

**Authors:** Arabinda Mishra, Feng Wang, Li Min Chen, John C. Gore

**Affiliations:** 1 Vanderbilt University Institute of Imaging Science, Vanderbilt University, Nashville, TN USA; 2 Department of Radiology and Radiological Sciences, Vanderbilt University Medical Center, Nashville, TN USA; 3 Department of Biomedical Engineering, Vanderbilt University, Nashville, TN USA

**Keywords:** Diseases of the nervous system, Sensorimotor processing, Somatosensory system

## Abstract

This study aims to evaluate how parameters derived from diffusion tensor imaging reflect axonal disruption and demyelination in specific white matter tracts within the spinal cord of squirrel monkeys following traumatic injuries, and their relationships to function and behavior. After a unilateral section of the dorsal white matter tract of the cervical spinal cord, we found that both lesioned dorsal and intact lateral tracts on the lesion side exhibited prominent disruptions in fiber orientation, integrity and myelination. The degrees of pathological changes were significantly more severe in segments below the lesion than above. The lateral tract on the opposite (non-injured) side was minimally affected by the injury. Over time, RD, FA, and AD values of the dorsal and lateral tracts on the injured side closely tracked measurements of the behavioral recovery. This unilateral section of the dorsal spinal tract provides a realistic model in which axonal disruption and demyelination occur together in the cord. Our data show that specific tract and segmental FA and RD values are sensitive to the effects of injury and reflect specific behavioral changes, indicating their potential as relevant indicators of recovery or for assessing treatment outcomes. These observations have translational value for guiding future studies of human subjects with spinal cord injuries.

## Introduction

Traumatic injuries to spinal cord (SCI) may impair sensory, motor and/or autonomic functions and compromise the quality of life^1^. Functional and behavioral impairments follow a cascade of events that are triggered by the initial insults to the spinal cord, but some functions often recover spontaneously over time^2,3^ by processes that are not well understood. Progress in developing effective therapies has been hindered partly by our inability to track and quantify injury associated pathological changes in different tracts of the cord, or to differentiate axon disruption and demyelination within the cord over time, in a segment and tract-specific manner. This inability reflects the poor sensitivity, specificity and spatial resolution of the imaging methods available. Here we demonstrate the potential role of diffusion tensor imaging (DTI) for characterizing changes in the diffusion properties of spinal cord white matter longitudinally. In particular we evaluated the relationships between changes in quantitative diffusion metrics within specific sensory (dorsal column) and motor (lateral column) white matter tracts after a well-localized, targeted injury, along with corresponding behavioral deficits in sensorimotor function and skilled hand uses in primates. The results suggest that quantitative DTI metrics may be useful biomarkers for assessing recovery from injury and the effects of interventions.

In primates, the spinal cord conducts fine touch (somatosensory) signals through ascending dorsal columns as well as lateral spino-thalamic pathways, whereas top-down descending motor control signals are carried by lateral and ventral columns, along with local neural circuits that regulate involuntary reflex actions. In previous studies, metrics derived from conventional T2- or T1-weighted images have proven relatively insensitive and non-specific for assessing disorders related to sensorimotor function. DTI acquires MRI signals in the presence of magnetic field gradients applied in multiple directions, which are sensitive to the displacements of water due to diffusion over a specified time. Numerous previous studies have used DTI to detect axonal injury and demyelination in both animal models, and human subjects, in e.g. traumatic brain injury and in disorders such as multiple sclerosis. Metrics derived from DTI (the axial (λ_||_ or AD) and radial diffusivity (λ_T_ or RD), fractional anisotropy (FA) and mean diffusivity (MD) have provided new insights into the relationships between injury induced disruptions in white matter microarchitecture and neurological impairments^4–16^. While DTI has been predominately used to study brain structure, it can also characterize and monitor pathological changes in the white matter of the spinal cord including the effects of traumatic injury^4,7,17–22^. DTI can quantitatively evaluate the microstructural properties, orientations, continuity and possibly myelination state of white matter fiber bundles, which is information that is beyond the reach of other imaging measurements^21–23^.

It has been widely recognized that pathological changes in the spinal cord are distance (to the center of injury) dependent^24,25^, so the ability to monitor and separate axon disruption and demyelination in different sensory and motor tracts, both at the injury level as well as in remote segments above and below an injury, would be clinically significant because each tract is engaged in functionally distinct but associated functions and behaviors. To date, most studies have examined DTI parameters at various segment levels (C1–C6) of the cervical cord^26,27^, where regional parametric changes over all the white matter in each segment were taken into consideration without consideration of whether changes are dependent on distance to the center of injury. Our recent multiparametric studies of monkey spines have shown that at high MRI field (e.g. 9.4 T), it is possible to clearly visualize and differentiate individual white matter tracts surrounding the grey matter of the cord and detect pathological changes in a region-specific and distance-dependent manner across multiple spinal segments^28–31^. For example, following a targeted unilateral injury to a dorsal column, we detected robust alterations in resting state functional connectivity (rsFC) between the dorsal and ventral horns of the cord, mainly at levels below the injury location, that recovered over time in line with improvements in behavioral function^32^. In the same set of monkeys, qMT (quantitative magnetization transfer) and CEST (chemical exchange saturation transfer) measures identified regional changes during recovery, in both white and gray matters, within and surrounding the injury and along the cord over time^30,31^. Using the same, spatially-constrained, unilateral dorsal column transection^3^, the present study aims to determine whether diffusion parameters derived from DTI are capable of detecting white matter changes and monitoring their progression during natural recovery in a tract- and segmental- specific manner.

Here we report our measurements of DTI-derived fractional anisotropy (FA), mean diffusivity (MD), radial diffusivity (RD), and axial diffusivity (AD) from regions of interest (ROIs) in different tracts and locations (injured and non-injured side, above and below the lesion), and compared the DTI parameters (1) between segments above and below the injury in the damaged dorsal column tract, (2) between dorsal column tracts on the injured versus the non-injured side, (3) between dorsal and lateral tracts on the injured side, (4) between dorsal and lateral tracts on the non-injured side, (5) across time to see which parameters change after injury, and (6) between pre-lesion and post-lesion conditions within the spinal cord. A major scientific objective is to determine the degree to which the recovery of hand sensorimotor impairment is linked to the recovery of fiber disruption and demyelination of the dorsal column tract white matter that is known to carry fine touch information to the brain and influence motor controls.

## Material and methods

### Animal preparation

Eight adult (6–8 years old) male squirrel monkeys *(Saimiri sciureus)* were included in this study. MRI acquisitions in the control group (n = 5, 13 runs) were performed in multiple sessions. A second group (n = 5, 15 runs) underwent unilateral dorsal column transections at an upper cervical spinal cord location (C5 level) and were scanned before and at different time points after injury, at different stages of recovery. In parallel, behavioral changes were also assessed before injury and after injury during recovery. The recovery periods ranged from 9–24 weeks post-injury. We stopped our data collection at different time points for each individual animal according to how their impaired behavior recovered. The pre- and post- lesion data were analyzed to derive the relationship between changes in DTI parameters and behavior following SCI.

For MRI data acquisitions, each animal was sedated with ketamine hydrochloride (10 mg/kg)/atropine (0.05 mg/kg) and maintained on mechanical ventilation with isoflurane anesthesia (0.5–1.1%) delivered in a 30:70 O_2_:N_2_O mixture. We have shown previously that light anesthesia with isoflurane allows the acquisition of high-quality image data with minimal motion artifacts and stable physiological signs. After intubation, each animal was placed in a custom-designed MR cradle and the neck secured with the imaging coil and padding. Saline was infused intravenously (3 ml/kg/h) throughout each imaging session to prevent dehydration and provide nutrients. SpO_2_ and heart rate (Nonin, Plymouth, MN), ECG, ET-CO_2_ (22–26 mmHg; Surgivet, Waukesha, WI), and respiratory pattern (SA instruments, Stony Brook, NY) were externally monitored. Rectal temperature was monitored (SA instruments) and maintained at 37.5–38.5 °C by means of a circulating water blanket (Gaymar Industries, Orchard Park, NY). Vital signs were monitored throughout the procedure from induction of anesthesia until full recovery. Further details about the procedures can be found in our previous publications^33,34^. After the end of data collection, each animal was euthanized and perfused. Post-mortem spinal cord tissue was extracted for histological evaluation. All procedures were approved by the Institutional Animal Care and Use Committee at Vanderbilt University. All experiments were performed in accordance with the NIH and relevant guidelines and regulations on laboratory animal care and use.

### MRI data acquisition

**Figure 1:**
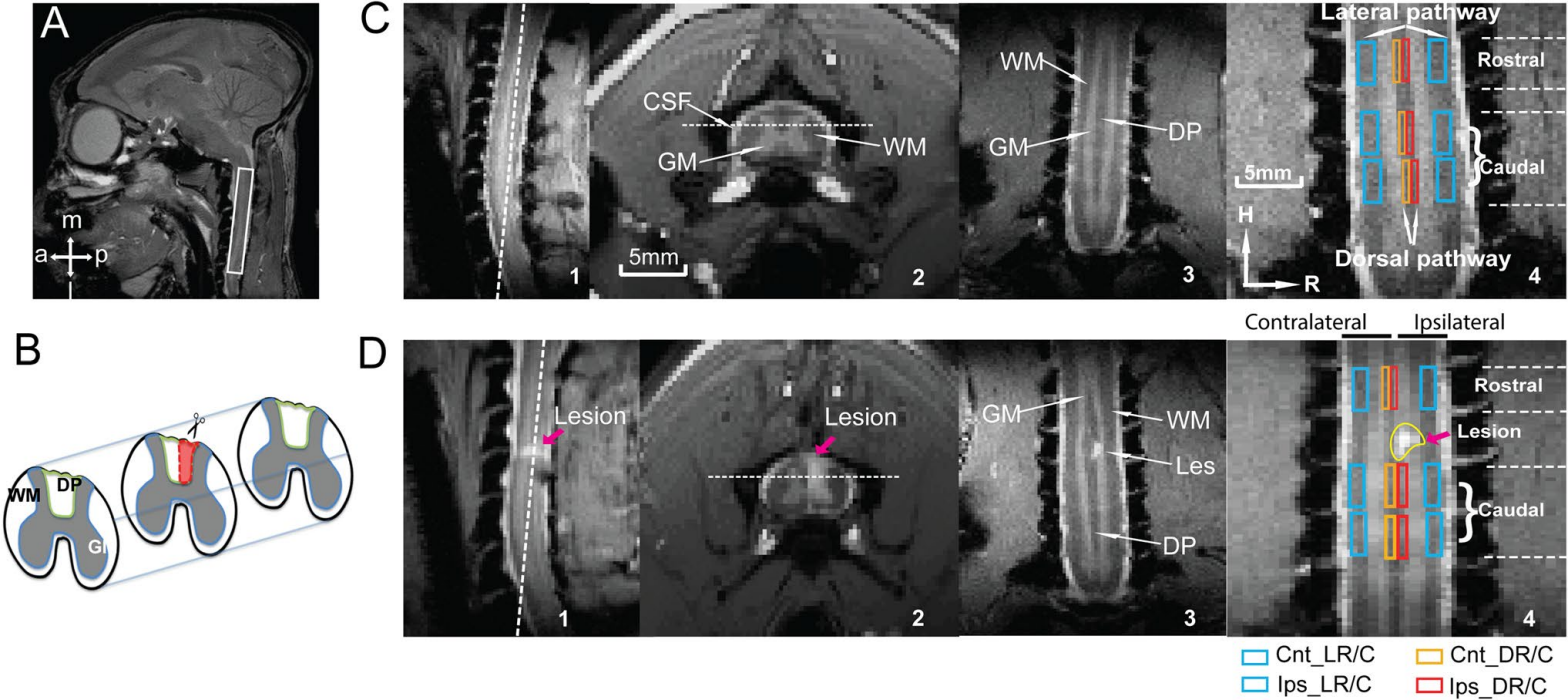
Illustration of spinal cord DTI and anatomical MRI data acquisitions and analyses in a representative squirrel monkey before and after SCI. (**A**) Sagittal view of a fast spin-echo T2-weighted monkey brain (white box shows the field of view for the spine data acquisition). (**B**) Schematic diagram of the targeted unilateral section of the dorsal column tract (DP) indicated by the outlined area (red) in the mid-axial slice. (**C**, **D**) White dotted lines on sagittal and axial images (1&2) refer to the locations of the coronal image shown in 3&4. White arrows on the axial and coronal images (2&3) indicate the white matter, gray matter (GM) and cerebrospinal fluid (CSF) of the spinal cord before and nine weeks after the section of the right dorsal column (**D**). C4 and D4 shows the corresponding ROIs identified on the rostral and caudal spinal segments of bilateral lateral (blue) and dorsal (orange/red) white matter tracts on the coronal slice (H: head, R: right). The lesion (indicated by red arrows) and high intensity cysts are visible on all three image plans in D. Nomenclature of lateral and dorsal (L/D) ROIs in the injured ipsilateral and non-injured contralateral sides rostral and caudal to the lesion is demonstrated using colored boxes below the panel D4. Ips_DR—injured dorsal tract rostral (above) to the injury location. Cnt_LC—non-injured lateral tract caudal (below) to the injury location.

All MRI images were acquired using a 9.4 T Agilent MRI spectrometer with a saddle shaped transmit receive surface coil positioned around the neck. The field of view was centered at C5 level, targeting the lesion (Fig. 1A). Eight coronal images with magnetization transfer contrast (MTC) were acquired using an MT-prepared gradient echo sequence (TR/TE = 220/3.24 ms; FOV = 32 × 32 mm^2^; matrix size = 128 × 128 (0.25 × 0.25 mm^2^ in plane resolution); slice thickness = 0.5 mm). A respiratory-gated, spin-echo echo-planar imaging protocol was implemented for DTI acquisitions using the following parameters: TR/TE = 3000/35 ms; duration of gradient pulse/diffusion time (δ/Δ) = 4/12 ms; amplitude = 19.96 gauss/cm; b-value = 1000 s/mm^2^; 30 gradient directions; isotropic spatial resolution 0.5 × 0.5 × 0.5 mm^3^; matrix size = 64 × 64 × 8; sampling bandwidth = 250 kHz; number of excitations = 16. The in-plane resolution of the MTC images was twice that of DTI images acquired using the same FOV and imaging orientation. A B_0_ map for each session was computed from two gradient echo images (ΔTE = 2 ms) to facilitate distortion correction on the DTI images.

### Spinal cord injury and behavioral assessment

Five animals underwent a 2 mm wide and 2 mm deep section of the dorsal column tract at C5 level on the side of the animal’s dominant hand. A detailed description of the surgical procedure was reported in our previous publications^35,36^. Each animal’s hand uses on a food reaching-grasping-retrieving task and in-cage activities were evaluated before and after the dorsal column section. Two behavioral scores of successful retrieval rate and number of flexes were measured for the task and were used later in linear regression analyses of DTI parameters. Typically, immediately after the injury, the SCI monkeys either avoided using their affected hand or used it with apparent functional deficits. For retrieving food, the success rate dropped, and the number of flexes needed increased. Over time, the frequency of hand use and ability to grasp increased, so the success rate increased, and the number of flexes decreased. Eventually there were no apparent abnormalities in routine in-cage activity at the end of the recovery period (for details of behavioral training and assessments see^3^). The total duration of the behavioral deficits and the speed of recovery varied across subjects, so decisions on when to scan each subject were based on behavioral improvements on an individual subject basis. For quantification purposes, we normalized the stages of recovery by plotting the curve of behavioral assessment and dividing the post-lesion duration into three phases: the beginning (recovery 1), middle (recovery 2) and end (recovery 3) of the recovery period. The recovery stage 2 was identified as 50% recovery period for comparison with DTI acquisitions. Therefore segregation/grouping of data and the number of measures were not based on the absolute time post injury. The pre-lesion and post-lesion measures at different stages were averaged across subjects for each region of interest (ROIs = 12, see Fig. 1) on the dorsal and lateral white matter tracts.

### ROI definition

The coronal MTC images provided adequate contrast to visualize the white matter^37^, gray matter (GM) and cerebrospinal fluid (CSF). Figure 1C1–3 show sagittal, axial and coronal views of a normal spinal cord. The relatively wide dorsal pathway (DP) seen in Fig. 1C4 between two dorsal gray matter horns was sub-divided into non-injured and injured dorsal tracts. Figure 1B shows a schematic view of unilateral transection of the dorsal pathway in a mid-axial slice (outlined in red). A signal void and a region of high intensity signal from cyst at the C4 level in the sagittal and coronal images are visible in Fig. 1D1–3 (9 weeks post-injury for a representative animal). In order to quantify regional changes in specific diffusion parameters, white matter ROIs were identified in the coronal slices for both dorsal and lateral tracts on both sides (four at each level), one segment above (rostral) and two segments below (caudal) the lesion (Fig. 1C4&D4). The unilateral section of the dorsal column tract provides a well-controlled, selective disruption of the sensory dorsal tract without interfering with the lateral motor pathways. The progression of pathology as a function of distance from the injury location along the cord was analyzed by quantifying changes in two selected diffusion parameters (FA and RD) (see Supplementary Figure for changes in all parameters) in three segments in different tracts.

### Calculation of diffusion parameters

**Figure 2:**
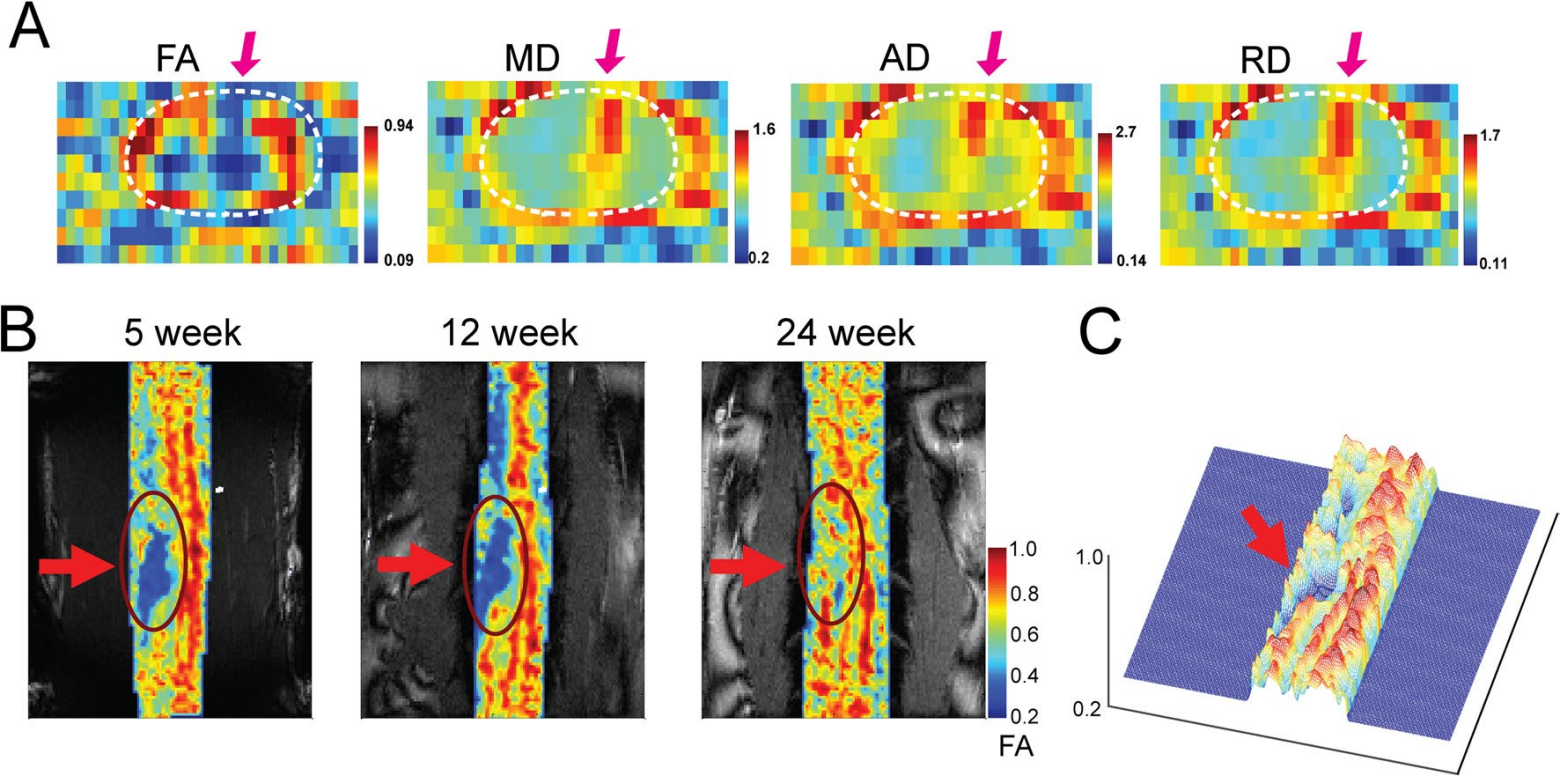
Representative post-injury diffusion parameter maps and the progression of FA maps. (**A**) Axial views of fractional anisotropy (FA), mean diffusivity (MD), axial diffusivity (AD) and radial diffusivity (RD) maps at three weeks post-injury overlaid on the anatomic MTC image. White dotted ellipses encircle the spinal cord—CSF interface in each map. (**B**) Coronal views of FA maps at three time points after a complete section of the right dorsal column. (**C**) 3-D illustration of FA profile shows the disrupted white matter tracts by injury at 5 weeks post injury.

Diffusion tensors were derived for each voxel using the thirty diffusion-weighted acquisitions from different orientations using CAMINO (*Camino.org.uk*). Artifact correction was performed using ACID (Artifact correction in diffusion MRI) taking the linear eddy current and motion artifacts into account^38,39^. A weighted linear fit was implemented to calculate the elements of the diffusion tensor^40^ and subsequently evaluated fractional anisotropy, mean, axial and radial diffusivities (FA, MD, AD and RD). The axial diffusivity AD describes the diffusion along the principal axis (λ_||_) of the white matter fibers whereas the radial diffusivity RD (λ_T_) quantifies how fast water diffuses across the axonal bundle and is smaller for healthy neuronal fibers in the white matter as diffusion is hindered or restricted. These quantities are defined as

1 $$FA = \sqrt {\frac{1}{2}} \frac{{\sqrt {\left( {\lambda _{1} - \lambda _{2} } \right)^{2} + \left( {\lambda _{2} - \lambda _{3} } \right)^{2} + \left( {\lambda _{3} - \lambda _{1} } \right)^{2} } }}{{\sqrt {\lambda _{1}^{2} + \lambda _{2}^{2} + \lambda _{3}^{2} } }}$$

2 $$AD = \lambda_{1}$$

3 $$MD = \frac{{\lambda _{1} + \lambda _{2} + \lambda _{3} }}{3}$$

4 $$RD = \frac{{\lambda _{2} + \lambda _{3} }}{2}$$

where λ denotes the principal eigenvalues of the diffusion tensor. AD and RD describe the diffusivities parallel and perpendicular to the axons under the assumption that the first eigenvector of the tensor corresponds to the orientation of the axons in the voxel. Diffusion parameter maps were overlaid on their corresponding anatomic images for display (e.g. Fig.  2).

### Statistical analysis of pre-versus post-lesion diffusion parameters

The post-injury changes in diffusion parameters in the lesion and ROIs in the white matter tracts were compared with control animals at corresponding locations. Changes along the dorsal and lateral tracts as a function of spatial distance from the injury location were evaluated using pre-injury measures as baselines. The significances of differences in parameters between stages of recoveries i.e., comparing beginning (recovery 1), middle (recovery 2) and end (recovery 3) of the recovery period, were calcuated using a non-parametric rank-sum test (Mann Whitney Wilcoxon test, MWW test) where *p* value < 0.05 or less (Bonferroni correction applied) was considered statistically significant. A behavioral recovery pattern was generated by averaging the two behavioral measures of success rate and the number of flexes (on a reversed scale) in the food grasping-and-retrieving task. Both success rate and the number of flexes were normalized to the pre-lesion baseline in each subject and then averaged for each time point (Fig. 7A, N = 5 animals). We performed linear regression analyses using the time courses of the diffusion parameters as independent variables measured over four stages (pre-lesion and three post-lesion) considering behavioral measures as dependent variables. The temporal variation of diffusion parameters extracted from eight ROIs and the lesion (Fig. 7A) were used as predictors of the group mean time courses of behavioral measures using a general linear model (GLM). We thereby derived the coefficient of determination (R^2^) to quantify how the temporal changes in DTI parameters predict the behavioral recovery. We performed least squares error estimation using a GLM and confirmed the normality and uniformity of the error distribution (homoscedasticity) prior to the analysis.

### Receiver operating characteristic analysis

Receiver operating characteristics^41,42^ were used to quantify the sensitivity, specificity and accuracy of the DTI parameters for distinguishing (a) between injury versus control sets of data, (b) between ROIs in the rostral and caudal segments at each stage of recovery and (c) between intact spinal cord and different stages of post-lesion recovery on various white matter tracts. The ROC curve plots the true positive rate (TPR, sensitivity) versus the false-positive rate (FPR, 1—specificity) at various threshold settings, which shows a tradeoff between true and false positive rates as the criterion for distinguishing conditions changes. The area under the curve (AUC) indicates the accuracy and overall diagnostic ability of each DTI parameter to discriminate the experimental groups or clusters.

### Histological analysis of post-mortem spinal cord tissue

At the end of the MRI acquisitions, spinal cord tissue was extracted for histological staining and analysis in four out of five monkeys who had undergone dorsal column lesions. We performed myelin staining to reveal myelin density^43,44^ along the cord long axis, crossing the lesion site and within the segment rostral to the lesion, and IBA1 (ionized calcium binding adaptor molecule 1) immunoreactivity to show activated microglia cells^45^.

## Results

### Changes in diffusion parameters in the injured dorsal column over time

**Figure 3:**
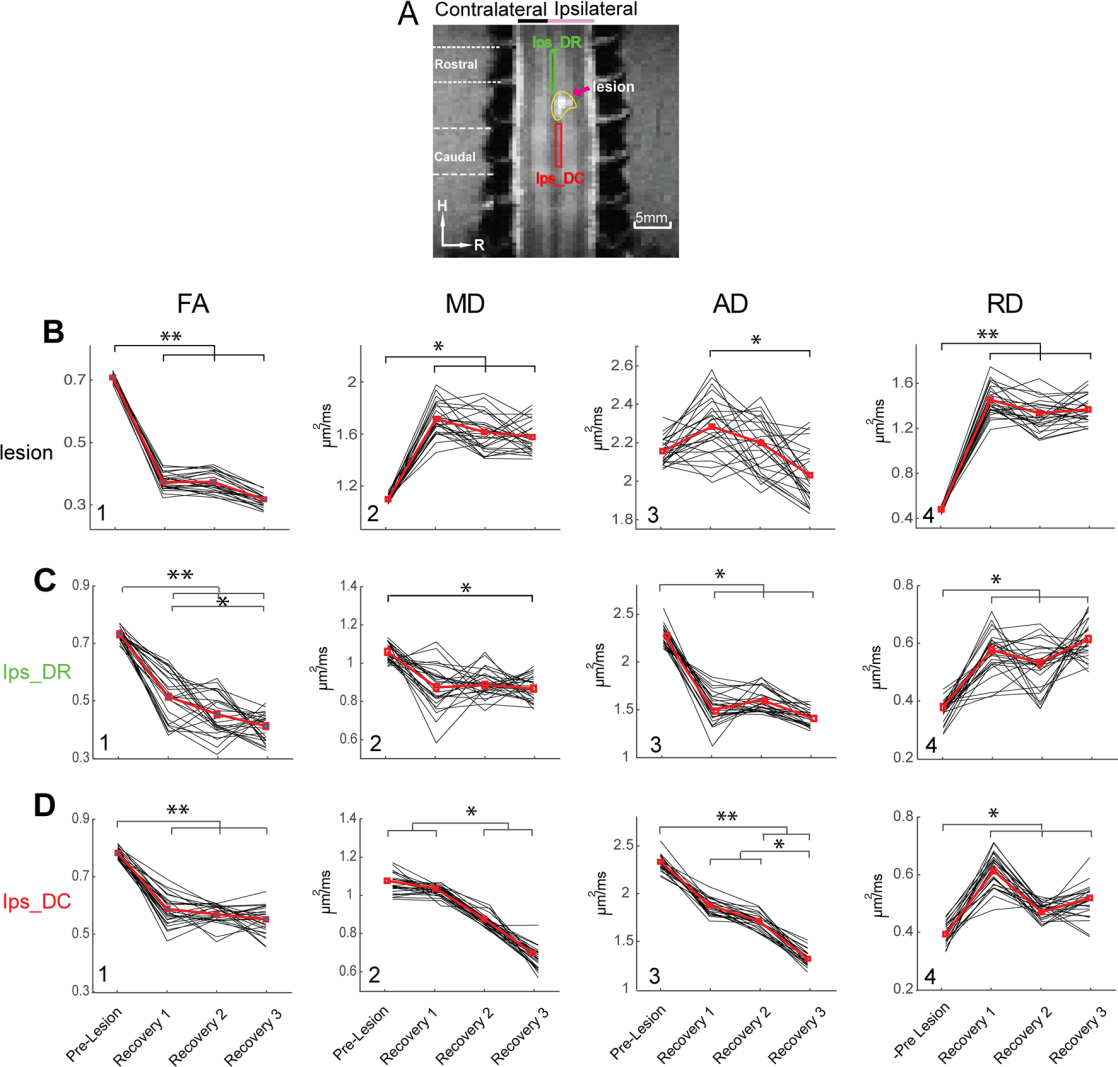
Group-average time courses of the diffusion parametric changes at the lesion and injured ipsilateral dorsal column over the period of recovery. (**A**) Identified rostral (green box) and caudal (red box) ROIs on lesioned dorsal tract and lesion (yellow contour) shown on the coronal MTC image. (**B**–**D**) Line plots of individual (black lines) and mean (bold red lines) FA, MD, AD and RD (1–4) values before (pre-lesion) and at three post-lesion recovery stages (1–3) at lesion (**B**), rostral (**C**) and caudal (**D**) ROIs. Statistical significance of difference between pre- and post-lesion parameters at various stages of recovery (N = 5 animals) are measured using MWW (Mann Whitney Wilcoxon) test (*p < 0.05; **p < 0.005, Bonferroni corrected). *p < 0.05; **p < 0.005, MWW test, Bonferroni corrected.

Figure 2A shows axial maps of FA, MD, AD and RD at 9-weeks post-injury in one representative monkey. The focal injury-induced changes are clearly visible (magenta arrows). The injury site was located in the right dorsal column tract as planned, and showed reduced FA but increased MD, AD and RD values. The lesion disrupted the dorsal tract running in the caudal to rostral direction. The MD, AD and RD values were larger at the lesion site compared to the surrounding white matter. Figure 2B shows another example of changes in FA over time during the recovery. In this case, the injury appeared to be bigger than that in the animal shown in Fig. 2A because a cyst was formed rostral to the lesion. Changes in FA from an early decrease can be clearly observed at three post injury time points. By post-injury week 24, the FA value of the injury region largely recovered but there was no clear white matter structure. For the injury group, compared to pre-injury measures at the lesion location, FA values significantly reduced (*p* < 0.005, Bonferroni corrected) immediately after the SCI (recovery stage 1) and remained low until the end of observations (Fig. 3B1). MD and RD values increased immediately after the injury and remained elevated (*p* < 0.05, 0.005 respectively, Fig. 3B2&B4) during the entire recovery period. A similar recovery trend was observed for AD values, though the increases following injury were not significant (Fig. 3B3). These observations confirm the impact and location of the targeted injury of the dorsal white matter tract, which chronically reduced the degree of anisotropic diffusion of water.

We also compared the FA, MD, AD, and RD values of the two spinal segments rostral (above) and caudal (below) the lesion site on the injured dorsal tract, before and at different times after injury. Figures 3C&D show the differences between pre- and post-lesion values for the rostral and caudal segments. After injury, FA, MD, and AD values dropped significantly. In the caudal segment, MD and AD values continued to decrease while corresponding values in the rostral segment remained level during recovery. Compared to the pre-lesion values, none of the injury-induced changes recovered by the end of study. Together, these findings indicate that a spatially focal lesion of the dorsal column tract introduced fairly constrained microstructural changes within the targeted white matter tract, and these structural changes did not recover by the end of recovery stage 3, when each monkey was able to perform the manual task behavior. The changes of DTI parameters in the rostral and caudal segments showed different time dependences. RD in both caudal and rostral segments was the only parameter that exhibited some degree of recovery. We observed the presence of a cyst in all cases.

### Changes in diffusion parameters in different locations over time

**Figure 4:**
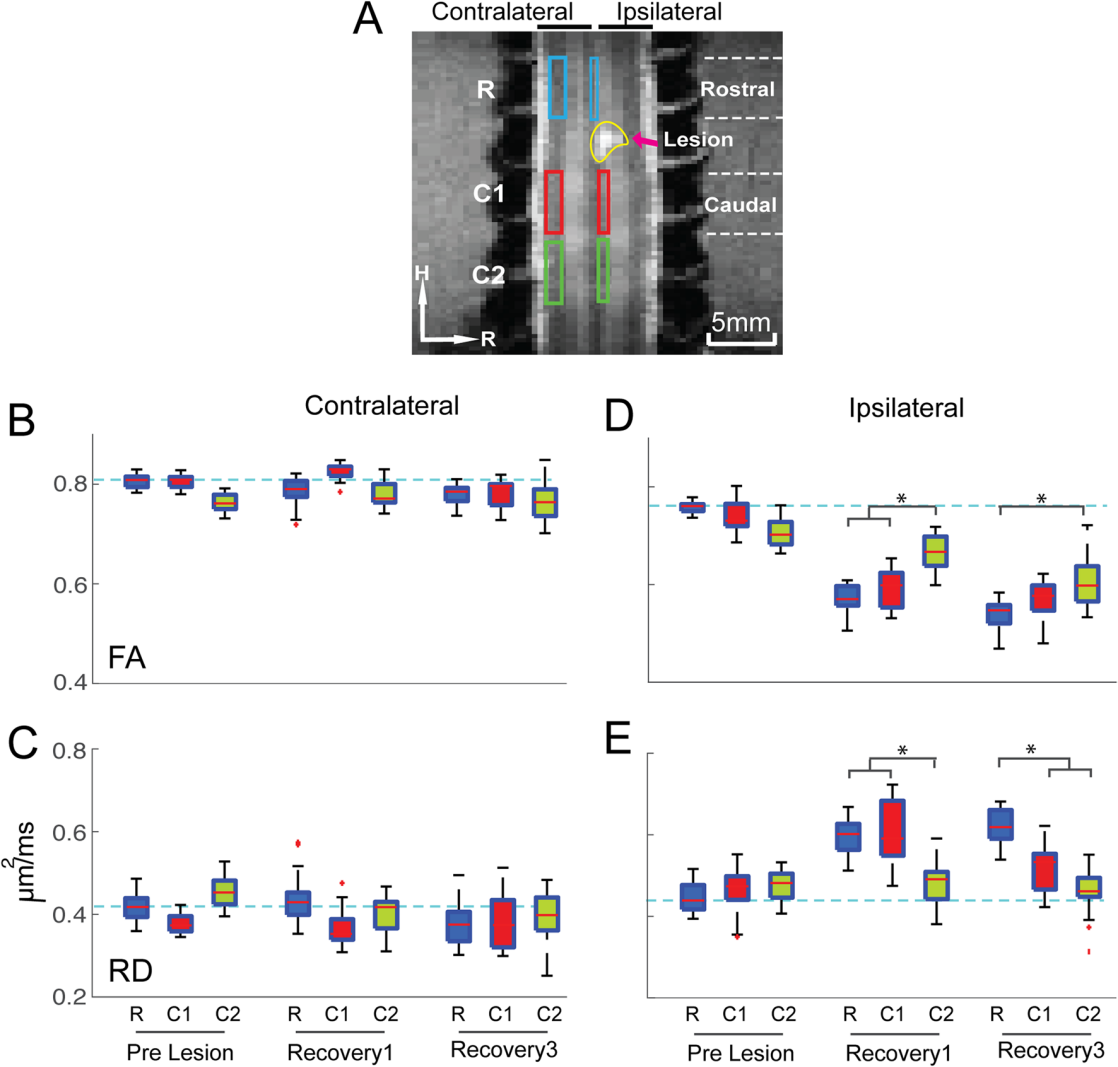
Distance-dependent temporal change in FA and RD measures on the injured ipsilateral dorsal column and non-injured contralateral lateral tracts. (**A**) Six ROIs selected for the ROI analysis with three on each tract. Each ROI covers one spinal segment. (**B**–**E**) Whisker Box plots of FA (**B**) and RD (**C**) value distributions in each of the three segments ROIs on two tracts (**A**, **B**: lateral, and **C**, **D**: dorsal) before injury (pre-Lesion) and at the beginning (recovery 1) and end (recovery 3) of the recovery. Statistical significance of difference measured using is presented by dark bars on top of the boxplots *p < 0.05, (MWW test, Bonferroni corrected).

Figure 4 shows the changes of DTI parameters (FA and RD) in the non-injured white matter compared with the lesioned pathway. Whisker boxplots of FA and RD from three segments of the lateral tract on the non-injured side showed negligible changes (Fig. 4B&C) before and after injury. In contrast, FA values in the two immediate segments (rostral (R-blue) and caudal (C1-red) ROIs) in the lesioned dorsal tract were significantly reduced (Fig. 4D) whereas RD values increased (Fig. 4E) at recovery stage 1 and 3 (stage 2 showed the same trend, see Supplementary Figure) compared to their pre-injury measures and those of the most distant segment C2 (green). This observation indicates that changes of FA and RD values are distance dependent, and that substantial changes occurred in the rostral and caudal segments adjoining the injury location on the dorsal tract.

### Distance dependent changes of DTI parameters in the injured versus non-injured side

**Figure 5:**
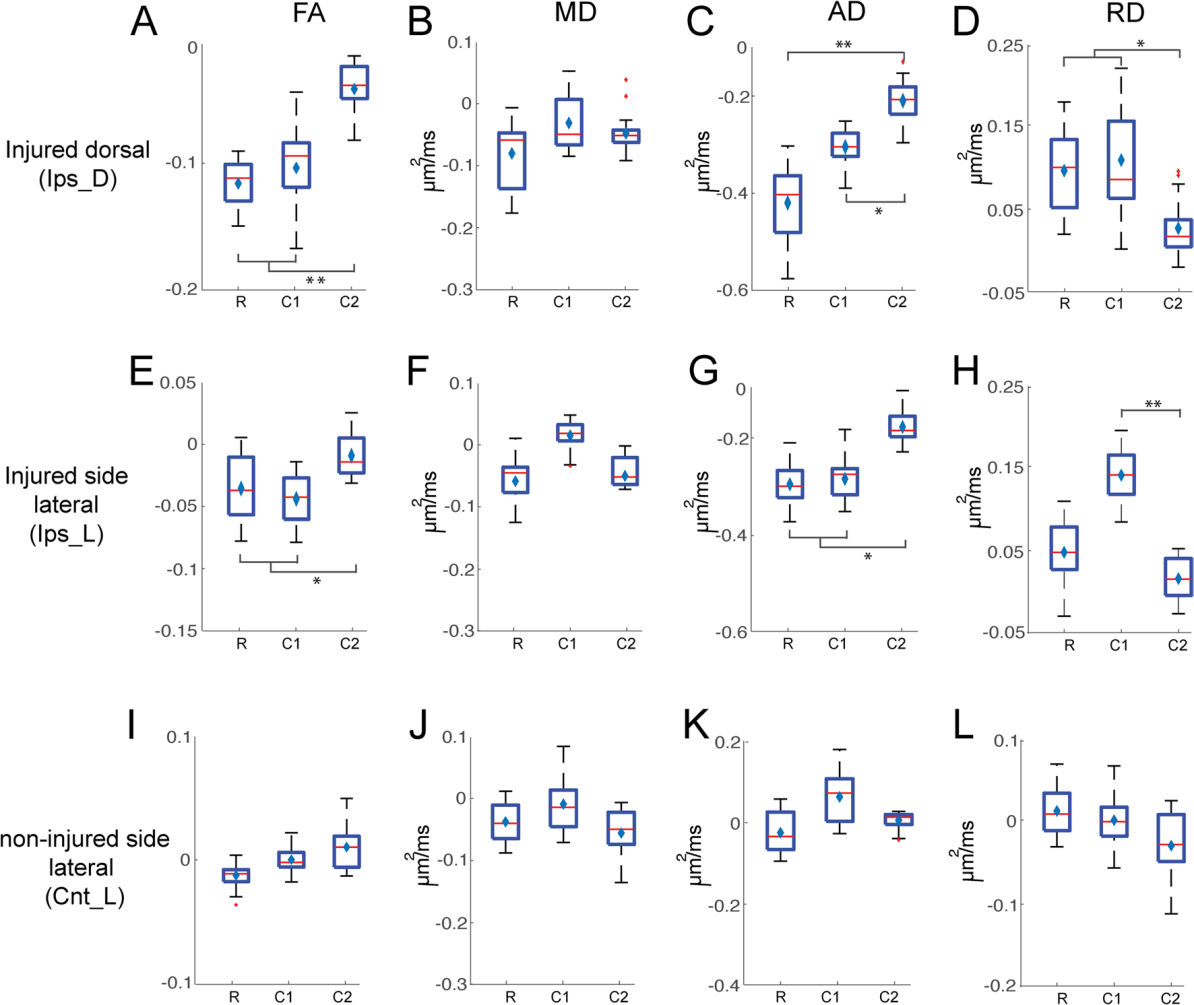
Segment-dependent changes of DTI parameters on three tracts after injury. Box plots of changes of FA, MD, AD, and RD values of the injure dorsal (**A**–**D**), intact lateral on the injured side (**E**–**H**) and lateral on the non-injured side (**I**–**L**) tracts, considering the pre-injury parameters as the baseline. *p < 0.05; **p < 0.005, MWW test, Bonferroni corrected). All post-injury DTI parameters (regardless of time points) were pulled together in this analysis.

Figure 5 illustrates the injury-induced changes of FA, MD, AD, and RD relative to their pre-lesion measures at each spinal segment regardless of recovery time. DTI parameters in the three segments of the non-injured lateral tract did not show significant changes (Fig. 5I–L). Similar to the previous findings (Fig. 4) along the lesioned dorsal track, FA, AD, and RD showed apparent changes in the two most immediate segments to the injury (Fig. 5A,C,D). The structural integrity of those white matter pathways which are close to the injury location were more severely affected after SCI. The disruption of white matter tract integrity measured by FA extended beyond one segment caudal to the injury segment (Fig. 4D).

### Classification and evaluation of pre- and post-injury DTI parameter changes using ROC

**Figure 6:**
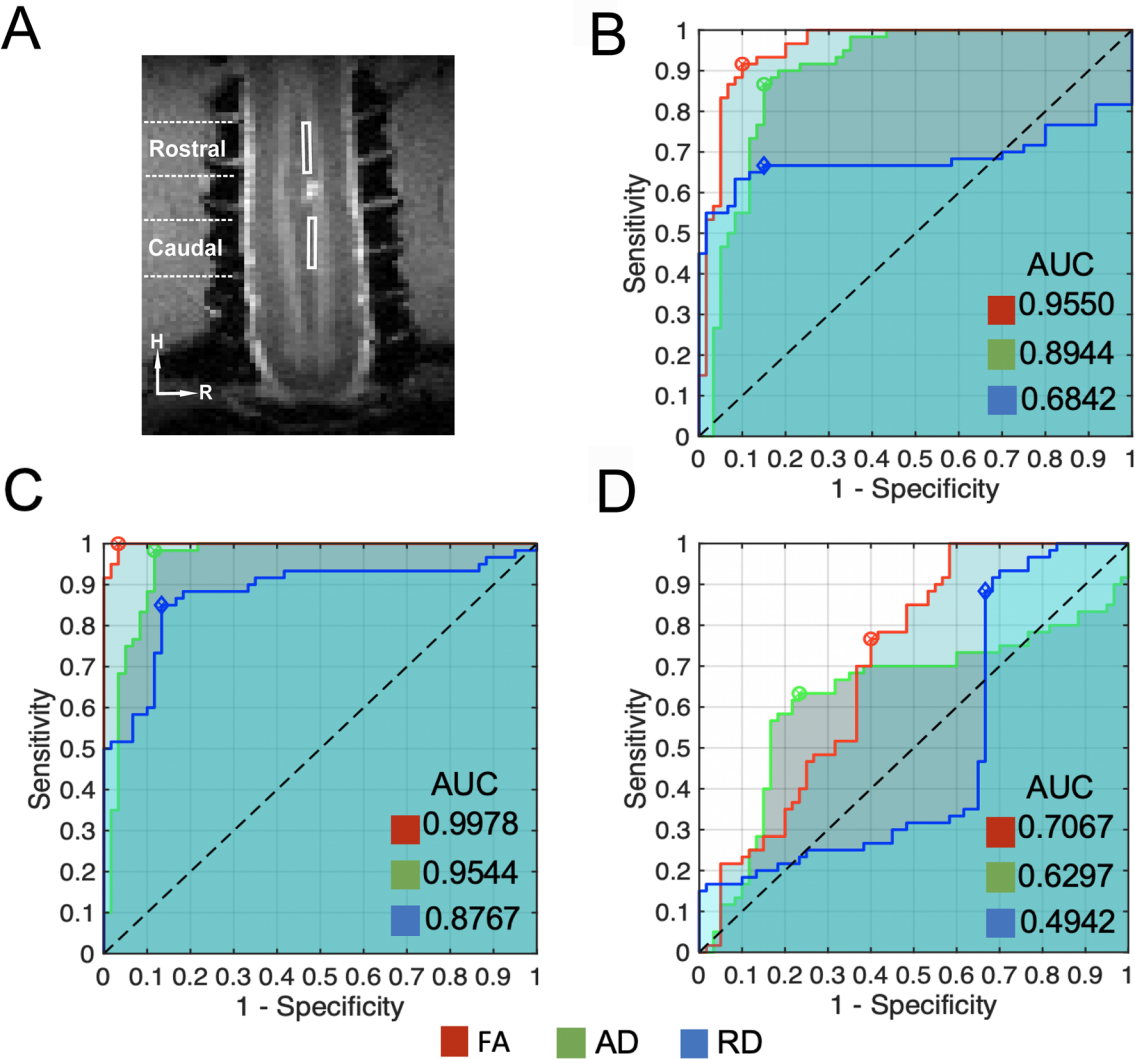
Accuracy of FA, AD, and RD values in discriminating lesioned and intact dorsal tract measured by ROC. (**A**) Identified ROIs on immediate rostral and caudal segments to the lesion (pink arrow). (**B**, **C**). ROC plots of true positive (TPR, sensitivity) versus false positive rate (FPR, 1—specificity) curves between pre-lesion and recovery stage 1 of the lesioned dorsal tract ROI rostral (**B**) and caudal (**C**) to the injury location. (**D**) ROC plots of the TPR and FPR relationships between the caudal and rostral ROIs on during recovery stage 1. The areas under the curve (AUC) for FA (red), AD (green) and RD (blue) are shown in each respective panel.

The ability of the DTI parameters to classify the pre- versus the post-injury conditions at various recovery stages, and between ROIs (Fig. 6A) at specific times, was measured by the AUC (area under curve). Figure 6B&C show the ROC plots of sensitivity (TPR) versus false positive rate (FPR) for FA (red), AD (green), and RD (blue) of the rostral (Fig. 6B) and caudal (Fig. 6C) segments of the lesioned dorsal tract, when distinguishing the pre- from the post-lesion recovery stage 1. Similarly, how well the above three parameters discriminate the spatial segments i.e., above and below the injury location at recovery stage 1 is shown in Fig. 6D. The FA, AD, and RD measures of both the caudal and rostral segments showed very high sensitivity, specificity, and accuracy in discriminating injured from intact pre-lesion conditions. The overall classification accuracy indexed by AUC shows that FA and AD, as compared to RD, classified the pre-lesion and post-lesion recovery stage 1 groups more accurately (AUC > 0.89).

FA was the most sensitive DTI measure whereas RD was fairly insensitive to the injury-induced demyelination and degeneration in the segment rostral to the lesion, as indicated by low AUCs (0.68 for rostral and 0.87 for caudal). The sensitivity, specificity, and accuracy of FA, RD, and AD values, however, were much lower in discriminating injured rostral versus caudal segments at all three recovery stages. The spatial classification parameters at recovery stage 1 (injured dorsal vs. caudal) shown in Fig. 6D were found to be smaller in comparison to the temporal parameters in Fig. 6B&C. MD was minimally sensitive to differences in both spatial (rostral versus caudal) and temporal (pre- and post- lesion) changes, and so was not included in the Figure plots. Together, FA and AD have high accuracy for classifying normal and lesioned spinal cord in all three post-lesion stages and for injured dorsal and caudal segments.

### Linear regression of temporal changes in diffusion parameters and behavioral changes during recovery

**Figure 7:**
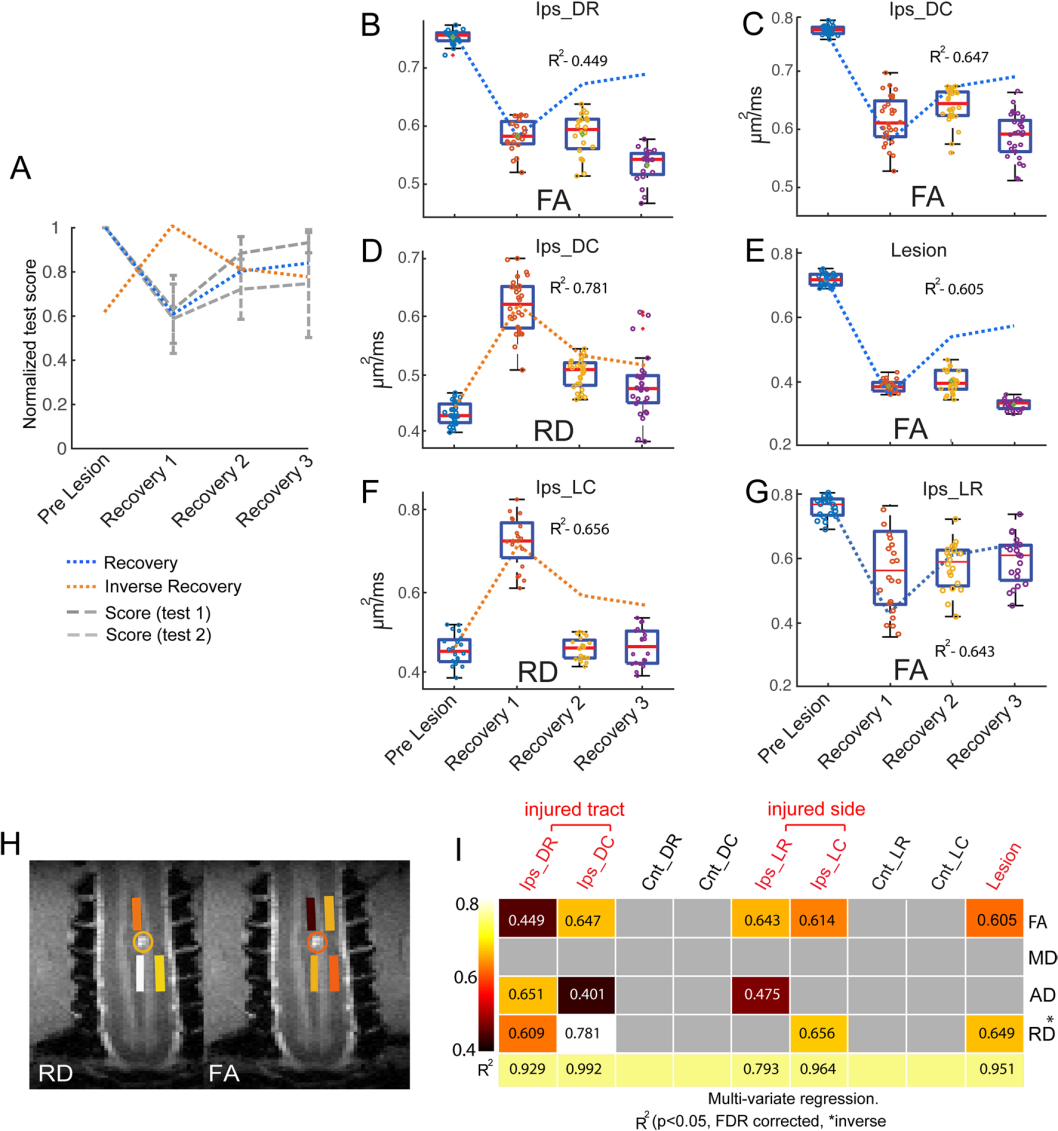
Linear regression analysis of DTI parameters and behavioral performance during recovery. (**A**) Normalized test score of behavioral performance before and at three stages during recovery. Score 1: successful rate. Score 2: number of flexes used for food retravel. Blue and orange dotted line refers to the model of recovery and inverse recovery in the regression analysis. (**B**–**G**) Boxplot shows the temporal change of diffusion parameters and corresponding R^2^ values for FA (B&C, rostral and caudal segments), RD (**D**) of injured dorsal tracts (caudal), RD (**F**) and FA (**G**) of intact lateral tracts on injured side, and FA of the lesion site (**E**) with the behavioral recovery curves (dotted line in the background). (**H**) ROIs of the injured dorsal and non-injured lateral tracts with color coded and thresholded R^2^ (coefficient of determination) values are overlayed on coronal MTC images to show their spatial relationships to the lesion site. (**I**) A matrix plot of color-coded and thresolded R^2^ values for all the ROIs tested. The same color code is used for both H&I.

We quantified the relationships between the temporal behavioral changes (Fig. 7A, mean ± standard deviation, blue and orange bold lines) and corresponding time courses of the diffusion parameters in nine ROIs (Fig. 7B–G are representative cases) including the lesion, using linear regression analysis. The regression analysis used the temporal variations of diffusion parameters as predictors to estimate the behavioral changes (normalized test scores prior to SCI, represented by the blue and orange dotted lines in Fig. 7A). The coefficient of determination (R^2^) measures how much variation in the behavioral changes could be explained by the diffusion parameters. Figure 7B–E show that changes in FA and RD values on the injured dorsal tract and the lesion site were good predictors of behavioral changes (R^2^ = 0.449 (Ips_DR), 0.647 (Ips_DC), 0.781 (Ips_DC), and 0.605 (lesion) and can be considered as reliable indicators of the behavioral recovery. Figure 7F&G show the relationships between distributions of RD and FA values of the non-injured lateral tract on the lesion side and measures of behavioral recovery. Figure 7H illustrates the specific locations of ROIs and their corresponding color-coded R^2^ values. The matrix plot shown in Fig. 7I summarizes the R^2^ values that measure statistically how closely the time courses of DTI parameters at each of the ROIs tracked the behavioral recovery curve. These ROIs include rostral and caudal areas on the lesioned dorsal tract (Ips_DR and Ips_DC), non-lesioned lateral tracts (Ips_LR and Ips_LC) on the injured and non-injured sides of the spinal cord, and the lesion itself. Figure 7H illustrates the specific locations of ROIs and their corresponding color-coded R^2^ values. Multivariate regression analyses using all four DTI parameters as predictors for each ROI resulted in increased R^2^ values (see the last row in Fig. 7I), indicating more accurate predictions when all four DTI parameters are considered. The DTI parameters of the caudal segment in the lesioned dorsal tract had the closest fitting and highest predictivity (R^2^ = 0.992) of behavioral recovery across all ROIs.

### Histological Validation of the white matter pathology

**Figure 8:**
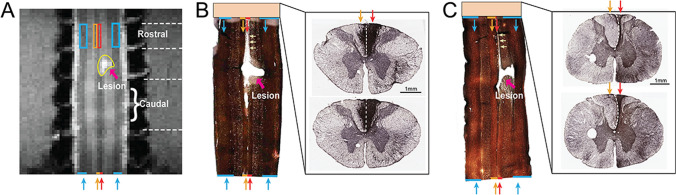
Histological evaluation of injured spinal cord in two representative subjects. (**A**) Coronal MTC image shows the ROIs identified on the white matter tracts (color rectangles) and the damaged tissue (yellow outline) at the lesion site. (**B**, **C**) myelin (left) and IBA1 (right) stains of the post-mortem tissue. Lateral tracts are indicated by blue arrows while the non-injured dorsal column tracts are indicated by orange arrows and injured tract is indicated by red arrows. Dotted white lines indicate the middle line between the left and right dorsal hemispheres. Yellow arrows show the tract regions that lost myelin.

We performed a histological analysis to document the underlying neuropathology and to provide a basis for interpreting the DTI findings. Figure 8A shows the corresponding MTC structural image of the case shown in Fig. 8B and the ROIs identified in each tract. Figures 8B&C show the myelin stain of coronal slices (left panels) across the injury center and glia IBA1 stain of axial slices (right panels) from the rostral segment of the spinal cord in two monkeys. On coronal myelin stains, significantly reduced myelin staining (light region) on the rostral sections of the lesioned dorsal column tracts (indicated by yellow arrows in Fig. 8B&C) were apparent in all cases. Light myelin staining indicates the demyelination of white matter tracts. The lesion-induced tissue cavity was apparent. There were also reduced myelin staining (light regions) around the lesion cavity. One noticeable difference between these two cases is the significant shrinkage or atrophy of the lesioned dorsal column tract in subject two (see the tract indicated by red arrows Fig. 8C). This atrophy was also apparent on the axial IBA1-stained slice of the cord segment rostral to the lesion (compare the black regions indicated by red arrows in the right images in Fig. 8B&C). Together, histological analysis revealed significant demyelination and tract atrophy of the injured dorsal column tract both rostral and caudal to the injury site. Pathological changes were more severe in the rostral segment and were more variable across subjects.

## Discussion

### The ability of DTI to monitor progression and recovery of specific white matter tract pathology after traumatic injury.

Diffusion tensor imaging has been widely used in clinical and preclinical studies to reveal and quantify white matter changes in disease conditions including spinal cord injury^41,46,47^. Here we demonstrated the capacity of high-resolution DTI to detect and monitor tract-specific white matter pathology in a targeted and spatially constrained dorsal column transection model in primates. We chose to use a targeted dorsal column lesion (DCL) model in monkeys because it disrupts axonal integrity and leads to degeneration and demyelination of fibers along its tract while in principle sparing other white matter tracts. By performing a region-of-interest analysis of DTI data obtained at different time points during recovery, we detected spatially constrained and distance-dependent changes in the integrity of white matter microstructure immediately after injury, and tracked their recovery over a period of up to 24 weeks, during which impaired behavior recovers in most of the monkeys. We found that caudal segments of the lesioned dorsal column tract below the injury showed the most prominent changes in at least three DTI measures (FA, AD, and RD). Various degrees of changes were also observed in the intact lateral tracts on the lesion side. We attribute these changes to the secondary injury associated with the initial traumatic injury to the dorsal tract. No apparent DTI parameter changes were observed in the spared dorsal and lateral tracts of the non-injured side of the spinal cord as anticipated. Together, these results show that when DTI data are acquired with high spatial resolution and signal-to-noise ratio, it is possible to detect and monitor white matter pathology in a regional and tract-specific manner. The FA and RD values of the segment caudal to the injury on the lesioned tract appear to be the most sensitive in detecting white matter pathology (axonal disruption and demyelination) and are most reliable indices of behavior recovery. The graded changes in the lesioned dorsal and non-lesioned lateral tracts underscore the sensitivity of the DTI measures for identifying different type of injuries. Our results have significant implications for preclinical and clinical studies because they offer a way to directly link specific sensorimotor behavioral deficits and recovery to specific white matter tract pathology with high spatial detail^11,48,49^. The differences of DTI parameters observed in rostral versus caudal segments indicate up-stream and down-stream white matter tracts may react differently to an insult and could provide valuable information to identify spinal cord sites for therapeutic interventions and provide objective indices of treatment outcomes.

### Regional and distance-dependent along the injured white matter tract

We also examined white matter changes at the injury site and surrounding spinal segments. We have shown previously that spinal tissues at the center of injury undergo dynamic changes in cell density and accumulations of liquid if a cyst is formed^29^. Again, here we showed that FA, MD, and RD values are also sensitive measures for evaluating the degree and spatial extent of the damaged spinal cord tissue at the injury center (Fig. 3). During recovery periods, however, only MD and AD showed time-dependent recoveries, whereas 8 weeks after injury all the animals showed varying degrees of behavioral recovery.

Beyond changes at the injury site, we also found that DTI parameters changed after injury in both rostral and caudal segments of the lesioned dorsal column tract. The significant reduction of FA above and below the injury segments is in line with previous studies and supports the idea that FA is the most sensitive measure of structural disruptions of white matter integrity. In our study, the injury introduced smaller changes in FA and AD values above and below the lesion in the dorsal column tract on the non-lesion side, even though it resides immediately adjacent to the lesioned tract (Fig. 3C). Compared to MD, values of RD, FA, and AD captured the recovery process of the lesioned dorsal tract both above (rostral) and below (caudal) lesion segments (Fig. 7B). As an indicator of myelination state, the sensitivity of RD values in detecting white matter pathology did not surpass FA. The MD was relatively less sensitive to changes induced by the injury, and thus has limited value as an imaging indicator of white matter pathology. Nevertheless, MD inside the lesion region increased substantially (Fig. 3A2) over the recovery period, which could be due to edema, accumulation of extracellular liquid, or increases in RD due to demyelination. It is worth mentioning that the sharp decrease in FA would also be influenced by increased RD. The post injury data, although not sampled uniformly over time, were grouped based on the behavioral improvements evaluated after the injury. Here we observed a strong association between the recovery in FA and RD values at the caudal segment of the lesioned tract with the recovery of impaired sensorimotor behavior at the group level. In part, we attribute the slightly different recovery trends of DTI parameters in the rostral versus caudal segment to the varying degrees of demyelination and degeneration. Histology (Fig. 8) showed that the rostral segment of the lesioned dorsal column underwent more severe demyelination and degeneration, an observation that is consistent with existing knowledge. Interestingly, our previous functional MRI studies revealed similar intersegmental differences in inter-horn resting state functional connectivity. Future studies that link the functional and structural changes could provide critical information about the potential interactions between spinal white and gray matter after SCI.

### The accuracy of ROC indices of DTI metrics for discriminating injuries in white matter tracts: implications for clinical application

ROC analysis is a popular and powerful method for evaluating the accuracy of medical diagnostic systems, including imaging biomarkers. The most desirable properties of ROC analysis are that the derived indexes of accuracy, in particular area under the curve (AUC), are not influenced by the use of arbitrarily chosen decision criteria or thresholds. AUC determines the inherent ability of a test to discriminate between disease and healthy populations, the injured versus non-injured spinal white matter tract segment groups in our case. In our study, the segment-based and tract -specific FA and RD values were highly accurate (AUC: 0.89–0.99) in classifying temporal changes (before and after injury at three different recovery stages) in each of the rostral or caudal segments. They are less accurate in discriminating injured rostral and caudal segments (AUC: 0.7 & 0.63, Fig. 6D). This finding indicates that injury-induced white matter tract neuropathology indexed by FA and RD values was not distinguishable between rostral and caudal segments. This observation is partially supported by the subsequent post-mortem histology (Fig. 8), in which various degrees of demyelination and degeneration were observed in segments both rostral and caudal to the injury. Here we showed that ROC derived AUC index of FA and RD values confirms these are accurate classifiers that can be used to assist diagnosis of injured spinal cord white matter tracts.

### Technical considerations for longitudinal monitoring of tract-specific white matter pathology

Longitudinal studies of spinal cord injury face at least three critical technical challenges: the accuracy of image alignment across imaging sessions (days), the repeatability of the ROIs defined for quantification, and inter-subject variability in the severity of injury and recovery process. Increased spatial resolution and enhanced gray-white matter contrast provide anatomical markers that can be used to mitigate these challenges. In our case, MTC images provided excellent white–gray matter contrast within the cord, distinct from its use in the brain. When we centered the imaging planes around the dorsal column and the lateral tracts, we were able to place the ROIs repeatedly at the same locations in each animal. We also spent extra time to position the neck to straighten the cord. This practice should also be feasible for human subjects. The high in-plane resolution allows precise ROI placements in specific white matter tracts and reduces potential partial volume effects. Finally, we realize our behavioral data normalization may not be ideal, which was used to demonstrate the direct relationships of measurable hand use to DTI parameters. We will test the predictivity of MRI metrics for each subject when we accumulate more SCI cases. Although quantitative evaluations of diffusion parameters^8,50–52^ and tractography^7,30,36,53–55^ of spinal cord pathology have been reported previously in both human subjects and animal models, those studies did not precisely quantify tract-specific changes in the dorsal and lateral tracts close to the injury location, along the cord and across spinal segments.

In summary, we explored the utility of parametric diffusion tensor imaging (DTI) for longitudinally monitoring white matter pathology of disruptions of axonal continuity, the integrity of white matter microstructure, and myelination state, over time in a tract-specific manner following traumatic spinal cord injury. We found DTI measures of FA, AD, and RD are sensitive in detecting longitudinal and distance-dependent changes in white matter fiber integrity and demyelination over time. These parameter changes associate very well with the behavioral recovery of skilled hand use, indicating their potential as imaging indicators for predicting behavioral recovery from injury in a monkey SCI model. Subsequent histological evaluation of the white matter myelin content confirmed that the lesioned dorsal column underwent substantial demyelination and degeneration processes while other intact tracts retained healthy myelination. DTI parameters are thus reliable indicators of white matter integrity and myelination status. These baseline measures could also serve as controls of spontaneous recovery for studies aimed at evaluating treatment outcomes. These observations have translational value for guiding future studies of humans with spinal cord injury.

## Supplementary information

Supplementary Information.
