# Supplementary material for: Longitudinal changes in DTI parameters of specific spinal white matter tracts correlate with behavior following spinal cord injury in monkeys
Source: Sci Rep. 2020 Oct 14;10:17316. doi: 10.1038/s41598-020-74234-2 (PMC7560889; doi:10.1038/s41598-020-74234-2)
Supplement: Supplementary file 1 — Supplementary Information. [file 41598_2020_74234_MOESM1_ESM.docx]

**Longitudinal changes in DTI parameters of specific spinal white matter tracts correlate with behavior following spinal cord injury in monkeys**

*Arabinda Mishra^1,2*^, Feng Wang^1,2^, Li Min Chen^1,2*^, John C. Gore^1,2,3^*

1. *Vanderbilt University Institute of Imaging Science, Vanderbilt University, Nashville, TN*
2. *Department of Radiology and Radiological Sciences, Vanderbilt University Medical Center*

*Nashville, TN*

1. *Department of Biomedical Engineering, Vanderbilt University, Nashville, TN*

*Running title: DTI metrics of traumatic injury to spinal white matter*

**Corresponding author*

Li Min Chen MD, PhD

Associate Professor

Department of Radiology and Radiological Sciences

Institute of Imaging Science

Vanderbilt University Medical Center

Nashville, TN 37232

Tel: 9367069

Limin.chen@vanderbilt.edu

**
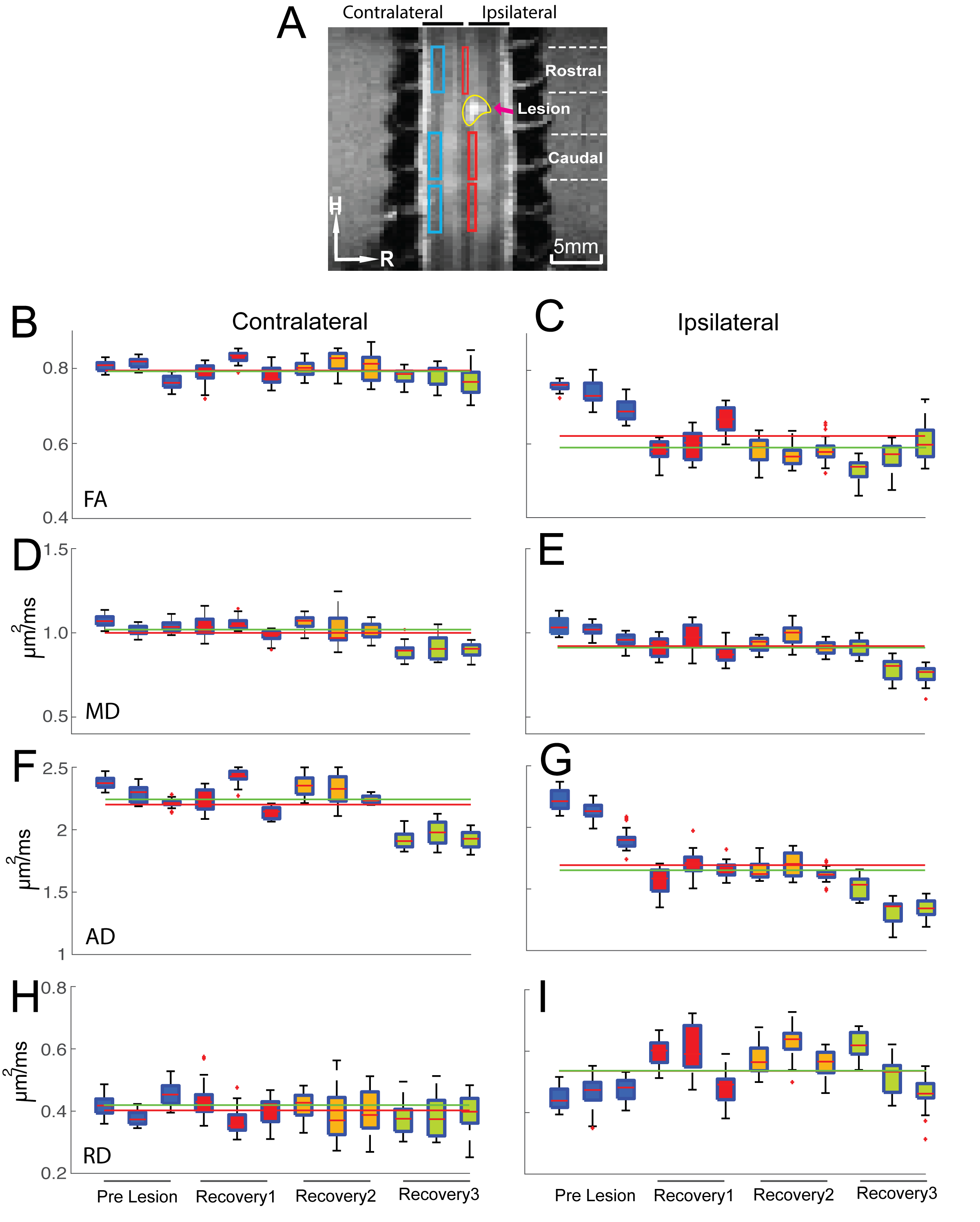
**

**Supplementary Figure. Variation of DTI parameters in each of the three ROIs in non-injured lateral and injured dorsal tracts before injury and at different stages during recovery.** (A) Locations of the ROIs on three spinal segments (one above and two below the lesion) on two white matter tracts: the intact lateral tract on the contralateral non-injured side of spinal cord, and the lesioned dorsal tract on the ipsilateral injured side of the cord. (B-I) Plots of DTI parameters (FA, MD, AD and RD) at pre- vs. post-lesion time points (recovery stages 1-3) for each of the three ROIs on the two white matter tracts. Short horizontal red bars in individual boxplots represent the median value of DTI parameters in individual ROIs. The long horizontal red and green lines refer to the median and mean values for each DTI parameter in all ROIs in the non-injured and injured tracts.
